# Transmission of methicillin-resistant *Staphylococcus aureus* in long-term care facilities and their related healthcare networks

**DOI:** 10.1186/s13073-016-0353-5

**Published:** 2016-10-03

**Authors:** Ewan M. Harrison, Catherine Ludden, Hayley J. Brodrick, Beth Blane, Gráinne Brennan, Dearbháile Morris, Francesc Coll, Sandra Reuter, Nicholas M. Brown, Mark A. Holmes, Brian O’Connell, Julian Parkhill, M. Estee Török, Martin Cormican, Sharon J. Peacock

**Affiliations:** 1 Department of Medicine, University of Cambridge, Addenbrooke’s Hospital, Box 157, Hills Road, Cambridge, CB2 0QQ UK; 2 London School of Hygiene & Tropical Medicine, Keppel Street, London, WC1E 7HT UK; 3 National MRSA Reference Laboratory, St. James’s Hospital, Dublin, Ireland; 4 Antimicrobial Resistance and Microbial Ecology (ARME) Group, School of Medicine, National University of Ireland, Galway, Ireland; 5 Cambridge University Hospitals NHS Foundation Trust, Hills Road, Cambridge, CB2 0QQ UK; 6 Public Health England, Clinical Microbiology and Public Health Laboratory, Addenbrooke’s Hospital, Box 236, Hills Road, Cambridge, CB2 0QW UK; 7 Department of Veterinary Medicine, University of Cambridge, Cambridge, UK; 8 Wellcome Trust Sanger Institute, Wellcome Trust Genome Campus, Hinxton Cambridge, UK

**Keywords:** MRSA, Whole-genome sequencing, Molecular epidemiology, Transmission, Long-term care facilities, Nursing home, *Staphylococcus aureus*

## Abstract

**Background:**

Long-term care facilities (LTCF) are potential reservoirs for methicillin-resistant *Staphylococcus aureus* (MRSA), control of which may reduce MRSA transmission and infection elsewhere in the healthcare system. Whole-genome sequencing (WGS) has been used successfully to understand MRSA epidemiology and transmission in hospitals and has the potential to identify transmission between these and LTCF.

**Methods:**

Two prospective observational studies of MRSA carriage were conducted in LTCF in England and Ireland. MRSA isolates were whole-genome sequenced and analyzed using established methods. Genomic data were available for MRSA isolated in the local healthcare systems (isolates submitted by hospitals and general practitioners).

**Results:**

We sequenced a total of 181 MRSA isolates from the two study sites. The majority of MRSA were multilocus sequence type (ST)22. WGS identified one likely transmission event between residents in the English LTCF and three putative transmission events in the Irish LTCF. WGS also identified closely related isolates present in colonized Irish residents and their immediate environment. Based on phylogenetic reconstruction, closely related MRSA clades were identified between the LTCF and their healthcare referral network, together with putative MRSA acquisition by LTCF residents during hospital admission.

**Conclusions:**

These data confirm that MRSA is transmitted between residents of LTCF and is both acquired and transmitted to others in referral hospitals and beyond. Our data present compelling evidence for the importance of environmental contamination in MRSA transmission, reinforcing the importance of environmental cleaning. The use of WGS in this study highlights the need to consider infection control in hospitals and community healthcare facilities as a continuum.

**Electronic supplementary material:**

The online version of this article (doi:10.1186/s13073-016-0353-5) contains supplementary material, which is available to authorized users.

## Background

Methicillin-resistant *Staphylococcus aureus* (MRSA) is a major cause of community- and healthcare-associated infections worldwide [1]. Infection typically occurs in individuals who are colonized with MRSA [2] and control measures to reduce infection rates largely revolve around the prevention of MRSA acquisition by non-carriers. In the United Kingdom (UK) and Ireland, rates of MRSA bacteremia have been reduced by more than 85 and 60 % in the past decade, respectively, following the introduction of mandatory surveillance and numerous infection control measures [3, 4]. This has led to calls for a zero tolerance approach to MRSA bacteremia [5]. Achieving further reductions requires consideration of residual reservoirs of MRSA carriage and activities associated with transmission [3].

Community-based long-term care facilities (LTCF) are an important reservoir for MRSA since rates of MRSA carriage may exceed 50 %, compared with a carriage rate of ~1.5 % in the general population in both the UK and USA [6, 7]. Frequent antibiotic consumption, indwelling devices, and the presence of chronic conditions in LTCF residents contribute to these higher carriage rates [8, 9]. Furthermore, the need to provide a homely environment combined with care of individuals with cognitive impairment poses challenges to effective infection control [10]. LTCF residents also have frequent contact with healthcare providers in hospitals and primary care, which represent opportunities for both onward transmission of MRSA and new acquisition events [11, 12].

Defining the extent to which LTCF contribute to the overall burden of MRSA acquisition and infection necessitates comparative genotyping of MRSA isolates from across healthcare networks. The use of standard genotyping methods such as multilocus sequence typing (MLST) has confirmed that MRSA in LTCF belong to a limited number of predominantly hospital-associated lineages [13, 14]. Existing methods do not, however, have sufficient resolution to delineate MRSA transmission within LTCF and between these and their associated healthcare network. Whole-genome sequencing (WGS) provides a solution to this problem and is being increasingly used to investigate the epidemiology and transmission of a range of bacterial pathogens [15–18]. Here, we describe the application of WGS to determine the frequency with which MRSA is transmitted between LTCF residents and their environment and is acquired and transmitted to others in referral hospitals and beyond.

## Methods

### Study design and sampling

We conducted two independent prospective observational studies of MRSA carriage in LTCF. The first was conducted in a 100-bed facility in Galway, Ireland between July 2012 and August 2013. The second was conducted in a 105-bed facility in Cambridge, UK over 27 weeks in 2014. Each LTCF consisted of four (Galway) or five (Cambridge) separate units to which residents were assigned based on cognitive impairment and nursing needs [19]. Data were collected from medical records and nursing care plans. This included demographics (age, gender, unit of residence), residence prior to admission to the study facility, a history of MRSA carriage or infection in the 12 months prior to enrolment and during the study period, and hospital contact over the same time scale. MRSA screening was performed at enrolment and then weekly (Cambridge) or quarterly (Galway) until the end of the study, participant discharge, or death. This was achieved by taking nasal (Galway) or multisite (nares and groin; Cambridge) screening swabs. No clinical MRSA infections were reported to the study investigators during the study period at either site.

### Environmental sampling

Environmental MRSA sampling was performed on four separate occasions as part of the Galway study, as previously described [19]. In brief, 9 months before the carriage study began, residents were transferred as a cohort from a pre-existing facility to a newly constructed residence. Multiple swabs of environmental surfaces were collected in communal and bedroom areas in the old and new facilities. This was done once in August 2011 in the old facility (denoted as environmental sample (ES) 1) and three times in the new facility (the study site), before moving in (ES2, August 2011) and before and after completion of the carriage study (ES3, November 2011 and ES4, August 2013, respectively) [19]. Environmental samples were processed and MRSA recovered from 94/270 (35 %) samples, as described previously [19], 24 of which were selected for WGS based on spread over time and genetic diversity as defined by previous MLST sequence type (ST) [19].

### Microbiology and bacterial isolates

In Cambridge, screening swabs were plated directly onto selective chromogenic MRSA medium (*Brilliance* MRSA2, Oxoid, Basingstoke, UK), incubated at 37 °C in air, and examined for MRSA growth after 24 h. *S. aureus* was identified using a latex agglutination test kit (Pastorex Staph Plus, Bio Rad Laboratories, Hemel Hempstead, UK). Isolates were confirmed as methicillin-resistant using cefoxitin, EUCAST methodology, and interpretive criteria [20]. Swabs taken in Galway were processed as described previously [19]. MRSA isolated from clinical samples by the diagnostic microbiology laboratory at Galway University Hospital between Oct 2011 and Nov 2011 (time of initial environmental screening) and Oct 2012 and Aug 2013 (time of patient study) were identified using the laboratory database. Thirty-seven isolates from 31 hospital patients in 2011 (n = 4), 2012 (n = 6), and 2013 (n = 27) were retrieved from frozen stock.

### Sequencing and analysis

Bacterial DNA was extracted and sequenced on an Illumina HiSeq2000 with 100-cycle paired-end runs. Sequence data have been submitted to the European Nucleotide Archive (ENA; http://www.ebi.ac.uk/ena) under the accession numbers listed in Additional file 1. Sequence data were assembled using the pipeline described in [21]. For each isolate the sequence reads were used to create multiple assemblies using VelvetOptimiser v2.2.5 [22] and Velvet v1.2 [23]. The assemblies were improved by scaffolding the best N50 and contigs using SSPACE [24] and sequence gaps filled using GapFiller [25]. Sequence types were determined from the assemblies using MLST check (https://github.com/sanger-pathogens/mlst_check), which was used to compare the assembled genomes against the MLST database for *S. aureus* (http://pubmlst.org/saureus/). SCC*mec* typing was carried out as previously described [26] using in silico PCR against velvet assemblies and published primers [27, 28]. Sequence reads were mapped to a relevant reference genome ENA ST22 (strain HO 5096 0412, accession number HE681097), ST5 (strain N315, accession number BA000018) or ST45 (strain CA347, accession number CP006044) using SMALT (http://www.sanger.ac.uk/science/tools/smalt-0) following default settings to identify single nucleotide polymorphisms (SNPs). SNPs located in mobile genetic elements were removed from the alignments and a maximum likelihood tree created using RAxML following default settings and 100 bootstrap replicates [29]. The ST22 isolates from Ireland and Cambridge were rooted using isolate MSSA476, as previously reported [30]. Trees with bootstraps values are presented in Additional files 2 and 3 for Galway and Cambridge, respectively. We also accessed genome sequence data for MRSA identified by the microbiology laboratory at Cambridge University Hospitals NHS Foundation Trust (Cambridge, UK) between April 2012 and April 2013 (2384 isolates from 1480 patients) (S. Peacock, personal communication). Ethical approval for this collection was given by the National Research Ethics Service (ref. 11/EE/0499), the National Information Governance Board Ethics and Confidentiality Committee (ref ECC 8-05 (h)/2011), and the Cambridge University Hospitals NHS Foundation Trust Research and Development Department (ref. A092428).

## Results

### Study participants

Sixty-four of 117 (55 %) residents at the Galway facility (Galway resident (GR)) and 45 of 90 (50 %) residents at the Cambridge facility (Cambridge resident (CR)) were recruited. Baseline characteristics of the two study groups are summarized in Table 1. No patients were known to have had an MRSA infection during the study. Seventeen participants in the Galway study died during the study and three participants were lost from the Cambridge study due to death (n = 2) or transfer to another facility (n = 1).

**Table 1:** Summary of study participants

Characteristic	Galway (n = 64)	Cambridge (n = 45)
Female (n (%))	30 (47 %)	29 (64 %)
Age (years), median (range, IQR)	80 (37–98, 9.25)	82 (40–104, 71–87)
Residence prior to admission (n (%))		
Home	39 (61 %)	7 (16 %)
Hospital	6 (9 %)	17 (38 %)
Other residential care	19 (30 %)	21 (47 %)
Hospital contact in 12 months before recruitment (n (%))	11 (17 %)	26 (58 %)
Hospital contact during study (n (%))	37 (58 %)	6 (14 %)
Known MRSA carriage or infection in 12-month period before recruitment (n (%))	9 (14 %)	3 (7 %)

*IQR* interquartile range

### MRSA carriage and phylogeny

**Fig. 1:**
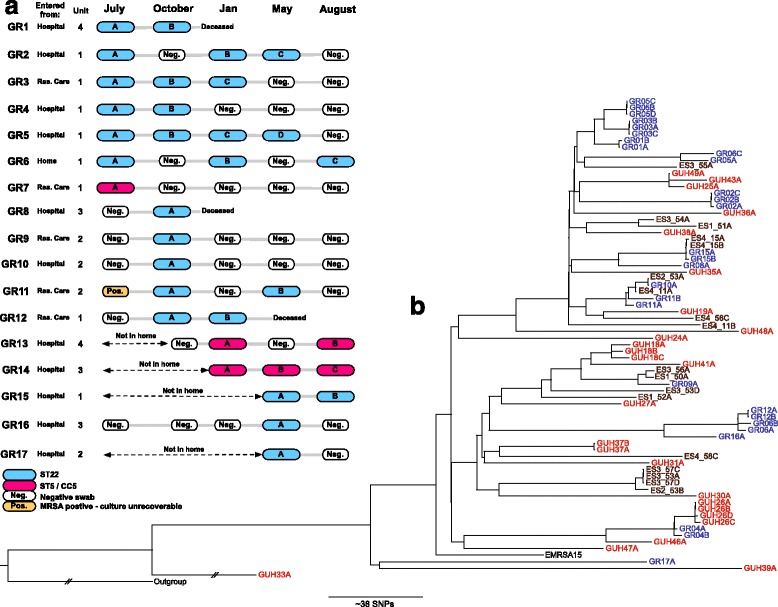
The epidemiology and phylogeny of MRSA in the Galway study facility. **a** Time line of positive and negative swabs. *Blue*, *pink*, and *orange shapes* representing ST22, ST5, and an MRSA-positive sample that could not be recovered on re-culture, respectively. **b** Maximum likelihood tree generated from core genome SNPs of ST22 MRSA isolates from the Galway LTCF residents (*blue*), environmental swabs (*black*), and isolates from Galway University Hospital (*red*). The outgroup is MSSA476. A tree with bootstrap values is shown in Additional file 1

In Galway, nasal swabs taken every three months for one year showed that 17 of 64 participants (27 %) were positive for MRSA in a total of 35 samples (Fig. 1a). The temporal pattern of carriage is shown in Fig. 1a. Sequence data were used to assign the ST to each isolate, which revealed the presence of two lineages: CC22 (n = 28) and CC5 (n = 6) (one isolate was unrecoverable for sequencing). A phylogenetic tree based on core genome SNPs of the ST22 isolates demonstrated multiple distinct clades (Fig. 1b). Two participants (GR05 and GR06) carrying ST22 had clade replacement over time or carried a mixed population. The clade identified from the first swab from GR05 (GR05A) was distinct from their later swabs (GR05B, C, and D) and the clade from the first two swabs from GR06 (GR06A and GR06B) was distinct from the isolate from their third swab (GR06C) (Fig. 1b). Three putative transmission events were identified based on genetic relatedness (<40 SNP; 40 SNPs is the maximum number of SNPs identified in sequencing of multiple colonies from a single individual [31]) and epidemiology. Two isolates from GR06 (GR06A and GR06B) and GR12 (GR12A and GR12B) were closely related with a maximum pairwise SNP distance of nine and both GR06 and GR12 were residents of the same unit (unit 1). Three more unit 1 residents (GR01, GR03, GR05) had isolates that resided in a cluster of highly related isolates that differed from each other by ~20 SNPs. A transmission event was also suggested for ST5 isolates; isolates from GR07 and GR13 had a maximum pairwise SNP distance of 15 (Additional file 4), the isolate from GR07 cultured 6 months before those from GR13 being basal in the cluster with GR13A and GR13B. However, these two residents lived on two different units located upstairs and downstairs in the home—unit 1 for GR07 and unit 4 for GR013—and GR07 was also immobile, indicating possible indirect transmission.

**Fig. 2:**
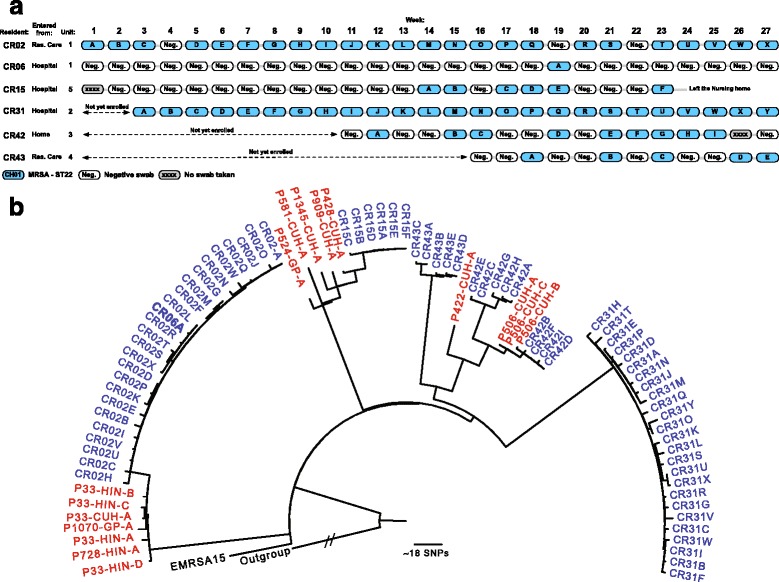
The epidemiology and phylogeny of MRSA in the Cambridge LTCF. **a** Time line of positive and negative swabs, with *blue shapes* representing ST22 isolates. **b** Maximum likelihood tree generated from core genome SNPs of MRSA isolates from the Cambridge LTCF (*blue*) and closely related isolates from Cambridge University Hospitals NHS Foundation Trust or Hinchingbrooke Health Care NHS Trust (*red*). The outgroup is MSSA476. A tree with bootstraps values is shown in Additional file 2

In Cambridge, 6 of 45 (14 %) participants were positive for MRSA in a total of 70 samples (Fig. 2a), all of which were ST22. Phylogenetic analysis demonstrated five distinct clades, each separated by at least 80 SNPs (Fig. 2b). Each participant was colonized by a distinct clade with the exception of CR02 and CR06, who carried the same clade. Furthermore, the single isolate from CR06 was nested in the cluster containing 24 isolates from CR02 with a two-SNP difference from the most closely related CR02 isolate, suggestive of a transmission event (Fig. 2b). This was further support by the fact that CR02 and CR06 both live in the same unit (unit 1). Evidence for acquisition of MRSA within the LTCF was observed for CR15, who became MRSA-positive after 12 negative screens (Fig 2). This individual carried a genetic lineage that was not closely related to other isolates; they did not have outside healthcare contact during the study and MRSA was presumably acquired from an unsampled carrier. Three participants (CR15, CR42, and CR43) were intermittently positive for MRSA, but each carried the same or highly related lineage either side of two consecutive negative swabs.

### Environmental contamination

The 24 environmental MRSA isolates from the four episodes of sampling that were sequenced belonged to ST22 (n = 18) or ST45 (n = 6). ST45 was not isolated from participants, yet was cultured from patient rooms (bedframes and lockers) in the old and new facilities (ES1, ES3) and shared areas (toilet flush, shower handle, and door handle in a common bathroom) in the new building (ES3), forming one cluster (Additional file 5). We assume that an unsampled resident, staff member, or visitor carried ST45. Comparison of the genetic relatedness of ST22 cultured from residents and the environment revealed several instances in which lineages were shared between the two. For example, GR15 carried MRSA that was also present in their immediate environment (ES4_15B; Fig. 1b). A striking example of environmental contamination was the finding that a carriage isolate from GR10 (GR10A) was highly related (five SNPs different) to an environmental isolate from the study facility before it was commissioned for use (ES2_53A). We also noted a cluster formed by MRSA carried by GR09 and isolated from both the old (ES1_50) and new facilities (ES3_56A) (Fig 1b). Eight genetically distinct ST22 isolates or clusters were isolated from the environment alone, which may have originated from unsampled residents, staff, or visitors.

### Relatedness of MRSA within healthcare networks

Next, we compared the genetic relatedness of MRSA from residents of the LTCF and MRSA isolated from elsewhere in the same healthcare network. In Galway, we obtained and sequenced 37 MRSA isolated from clinical samples taken from hospital in-patients at Galway University Hospital (GUH). These were assigned to nine different STs: ST22 (n = 25), a single locus variant of ST22 (n = 1), ST5 (n = 1), a single locus variant of ST5 (n = 1), ST779 (n = 4), ST8 (n = 2), ST45 (n = 1), ST88 (n = 1), and ST1 (n = 1). The 27 isolates with STs represented in both the hospital and LTCF carriage collections (CC22 and CC5) were included in further phylogenetic analysis; the hospital isolates were cultured from 21 patients with bacteremia (n = 17), deep site infection (n = 3), or a wound surface swab (n = 1). The phylogeny of MRSA genomes from the two settings indicated distinct bacterial populations, with two exceptions. Carriage isolates from GR04 and an isolate cultured from a hospital patient (GUH26, Jan 2013) were highly related (ten SNPs different) (Fig. 1b). Resident GR04 was admitted to GUH in the year before the carriage study started, suggesting initial acquisition at the hospital or onward transmission to others at that time (Fig. 1b). ST5 isolates from residents GR07 and GR13 were also related to an isolate from GUH34 (25 SNPs different; Additional file 4). Both GR07 and GR13 were admitted to GUH in the year before the study began.

A comparative analysis was also performed between carriage isolates from the Cambridge LTCF and an extensive, unbiased collection of all ST22 MRSA cultured at the diagnostic microbiology laboratory at Cambridge University Hospitals NHS Foundation Trust (CUH) from April 2012 to April 2013. This laboratory receives samples from three hospitals (CUH and Hinchingbrooke Hospital (HIN), and Papworth hospital) and GPs in the region. We identified 1132 CC22 isolates from this collection, which were combined in a phylogenetic tree with the Cambridge LTCF isolates, and used a cutoff of 50 SNPs (10 SNPs greater than the maximum number of 40 SNPs, found with isolates from a single individual [31–33]) to identify the most closely related MRSA from the diagnostic laboratory. This identified 16 MRSA isolates from ten patients at the CUH (n = 7), HIN (n = 2), and GPs’ practices (n = 2), which were related to carriage isolates from residents CR02, CR06, CR15, and CR42 (Fig. 2b). A case note review revealed that hospital patient CUH506 and resident CR42 were the same person. Isolation of their hospital strain occurred 18 months prior to detection of carriage during the study, suggesting that CR42 was admitted to the LTCF carrying this clade. A link between resident CR02 and a cluster of isolates from patient (P)33 and P728 was explained by multistep transmission events (Fig. 2b). CR02 and P33 had both previously resided in the same LTCF (different to the study site), where a presumed transmission event took place. P33, P728, and P1070 were all admitted to HIN during 2012, strongly indicating that this clone was circulating in that hospital. Even though CR15 had no hospital contact during the study period, their isolates (A–F) were closely related to an isolate from P428 who died in CUH in July 2012 (Fig. 2b). Two of the four isolates basal to the isolate from P428 were from two other patients (P581 and P524), who along with P428 were residents in another Cambridge LTCF.

## Discussion

Our study provides a genome-based view of MRSA carriage and transmission within LTCF and between these and referral hospitals. Our findings are consistent with high rates of MRSA carriage compared with hospital populations, on-going MRSA transmission between LTCF residents, and acquisition and onward transmission of MRSA during hospital admission. Our study also provides further evidence to suggest that the environment might represent an important MRSA transmission pathway. Rates of MRSA carriage in both LTCF were comparable to previously reported prevalence rates of 23.3 % in Northern Ireland (95 % confidence interval (CI) 18.8–27.7 %) and 19–22 % in Leeds, UK [14, 34]. The predominance of ST22 in both LTCF is consistent with previous studies in UK and Irish healthcare settings [30, 35]. Delineation of numerous ST22 clades in each facility is also consistent with previous reports of the discriminatory power of WGS [15, 17, 33].

WGS data provided strong evidence for on-going MRSA transmission within the study LTCF and hospitals. This was particularly observed in the Irish facility, in which seven residents shared closely related isolates with other residents. We noted that five of the six MRSA colonized Cambridge participants and 16 of the 17 colonized Galway participants had been in hospital in the previous year, confirming the potential for onward transmission and indicating an important infection control target. WGS identified that a Cambridge resident (CR42) carried an ST22 clade that was isolated during two hospital admissions and associated with infection approximately 18 months previously, suggesting ineffective MRSA decolonization protocols in known MRSA carriers. We found evidence for putative MRSA acquisition during hospital admission in the Galway study, in that three residents were hospitalized in the previous year and subsequently were colonized with MRSA clones found to be circulating in GUH.

We identified multiple instances where MRSA carriage in an individual was highly related to environmental MRSA from their single room or communal areas, as has been reported by WGS-based studies in hospitals [36, 37]. Eight ST22 clades and four ST45 clades were identified from the environment alone, which may be attributable to carriage of these lineages by unsampled residents or staff. ST22 has been reported to survive for weeks on abiotic surfaces and has been identified in hospital surfaces and on public buses [38, 39]. Additionally, both ST45 and the ST8 clone USA300 have been shown to play a role in environmental contamination and household transmission [40, 41]. Taken together, this suggests that the environment may be an important source for MRSA transmission and a barrier to effective infection control in LTCF, which is consistent with previous studies showing that cleaning can reduce environmental MRSA contamination and infection rates in hospitals [42].

We found no direct evidence for the introduction of MRSA into hospitals by our study participants, which might be expected given the study design, although the relatedness of study facility and hospital MRSA populations indicates that these are shared. We also captured the complexity of MRSA acquisition and transmission across healthcare networks, as highlighted by the transmission network between a Cambridge resident (CR02) and a cluster of hospital isolates from individuals who had shared a different community-based care home (P33) and had been admitted to the same hospital (P33, P728, and P1070). This putative transmission network exemplifies the complex inter-connectivity of hospitals and community-based care facilities. A similar epidemiology picture was observed for vancomycin-resistant *Enterococcus faecium* in the same Cambridge LTCF [43], demonstrating that the overlapping epidemiology between hospital and LTCF is likely common to most non-nosocomial pathogens.

We acknowledge several study limitations. Some participants carried different ST22 lineages over time, representing possible clade replacement or carriage of more than one lineage or clade. Sequencing was based on DNA extracted from a pure culture of a single colony. Serial sampling of individuals partially addresses this bias, but distinguishing between these two possibilities requires sequencing of multiple independent colonies from the primary culture plate [33]. Studies using both *spa*-typing and WGS have confirmed carriage of more than one strain [33, 44], although the frequency with which this occurs in different settings is not known. Furthermore, only the nares were sampled in the Galway study and the nares and groin in the Cambridge study, thus excluding MRSA carriers solely colonized at other body sites [45]. A further limitation is that only approximately 50 % of residents were recruited and sampled in both study sites and staff members were not screened, reducing the power to detect transmission. Many participants in both studies were immobile and understanding the pattern of staff MRSA carriage may shed light on the contribution of healthcare workers to the transmission dynamics of MRSA in LTCF.

## Conclusions

Our study confirms that LTCF are reservoirs for MRSA strains that are closely related to hospital strains of MRSA, the comparable results from two different geographical settings adding weight to these findings. This provides further evidence for the need for infection control policies that jointly consider hospitals and community healthcare facilities.

## Additional files

Additional file 1: Isolates and associated metadata included in this study. (XLSX 47 kb) Additional file 2: Phylogeny of CC22 MRSA in the Galway study facility with bootstrap values. Bootstrap values for branches are shown in *grey*. (PDF 197 kb) Additional file 3: Phylogeny of CC22 MRSA in the Cambridge LTCF with bootstrap values. Bootstrap values for branches are shown in *grey*. (PDF 224 kb) Additional file 4: Phylogeny of CC5 MRSA in the Galway study. Maximum likelihood tree generated from core genome SNPs of CC5 MRSA isolates from the Galway LTCF residents (*blue*) and isolates from Galway University Hospital (*red*). Bootstrap values for branches are shown in *grey*. (PDF 266 kb) Additional file 5: Phylogeny of ST45 MRSA in the Galway study. a Maximum likelihood tree generated from core genome SNPs of ST45 MRSA isolates from the Galway LTCF environment (*black*) and the isolate from Galway University Hospital (*red*). b Zoomed in view of the isolates from the Galway LTCF. Bootstrap values for branches are shown in *grey*. (PDF 23 kb)

## Abbreviations

CR: Cambridge resident
CUH: Cambridge University Hospitals NHS Foundation Trust
ENA: European Nucleotide Archive
ES: Environmental sample
GP: General practitioner
GR: Galway resident
GUH: Galway University Hospital
HIN: Hinchingbrooke Hospital
LTCF: Long-term care facility
MLST: Multilocus sequence typing
MRSA: Methicillin-resistant *Staphylococcus aureus*
P: Patient
SNP: Single nucleotide polymorphism
ST: Multilocus sequence type
WGS: Whole-genome sequencing.
