# Supplementary figures and images for: Transmission of methicillin-resistant Staphylococcus aureus in long-term care facilities and their related healthcare networks
Source: Genome Med. 2016 Oct 3;8:102. doi: 10.1186/s13073-016-0353-5 (PMC5048656; doi:10.1186/s13073-016-0353-5)

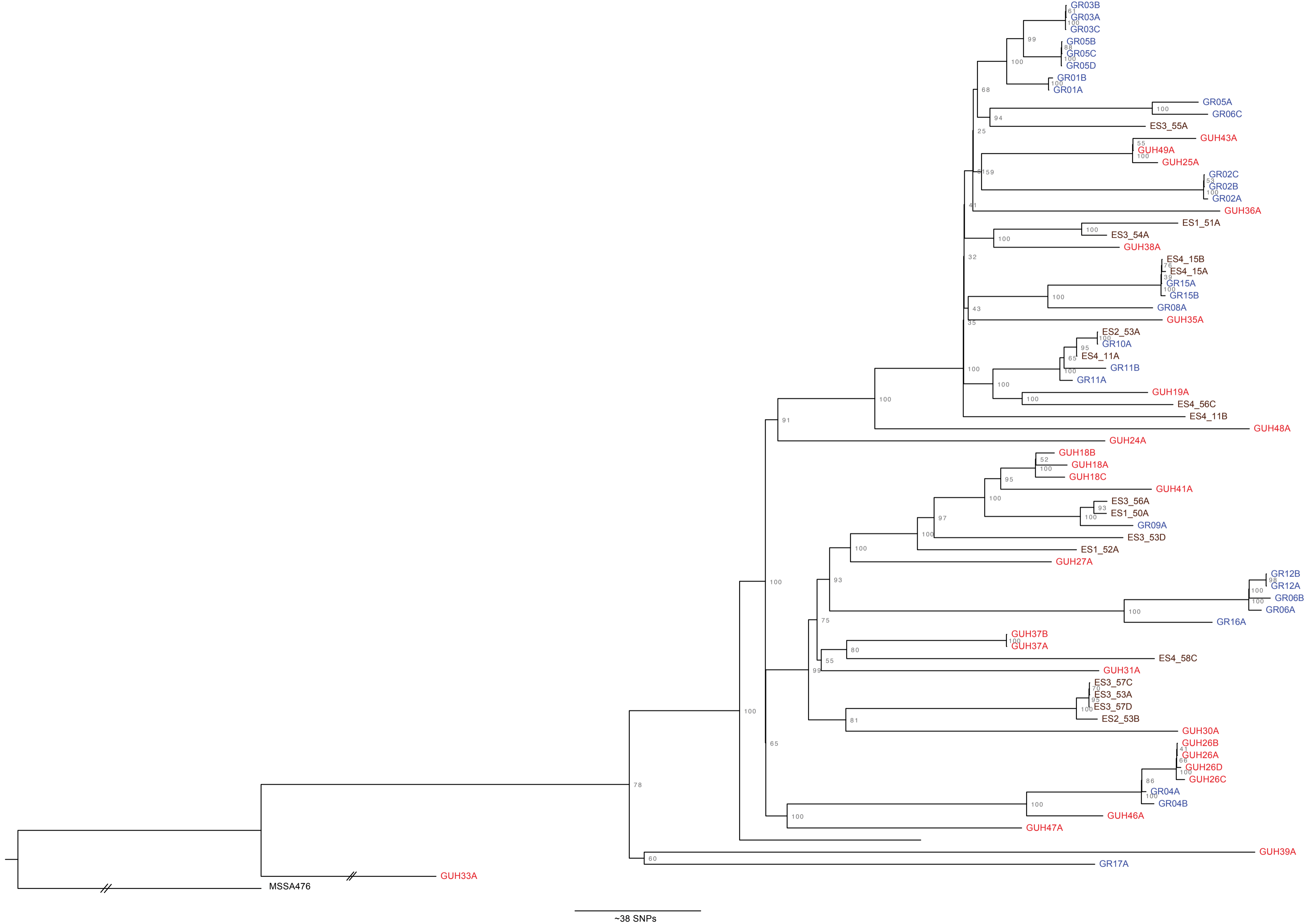

Supplement: Additional file 2: — Phylogeny of CC22 MRSA in the Galway study facility with bootstrap values. Bootstrap values for branches are shown in grey. (PDF 197 kb) [file 13073_2016_353_MOESM2_ESM.pdf]

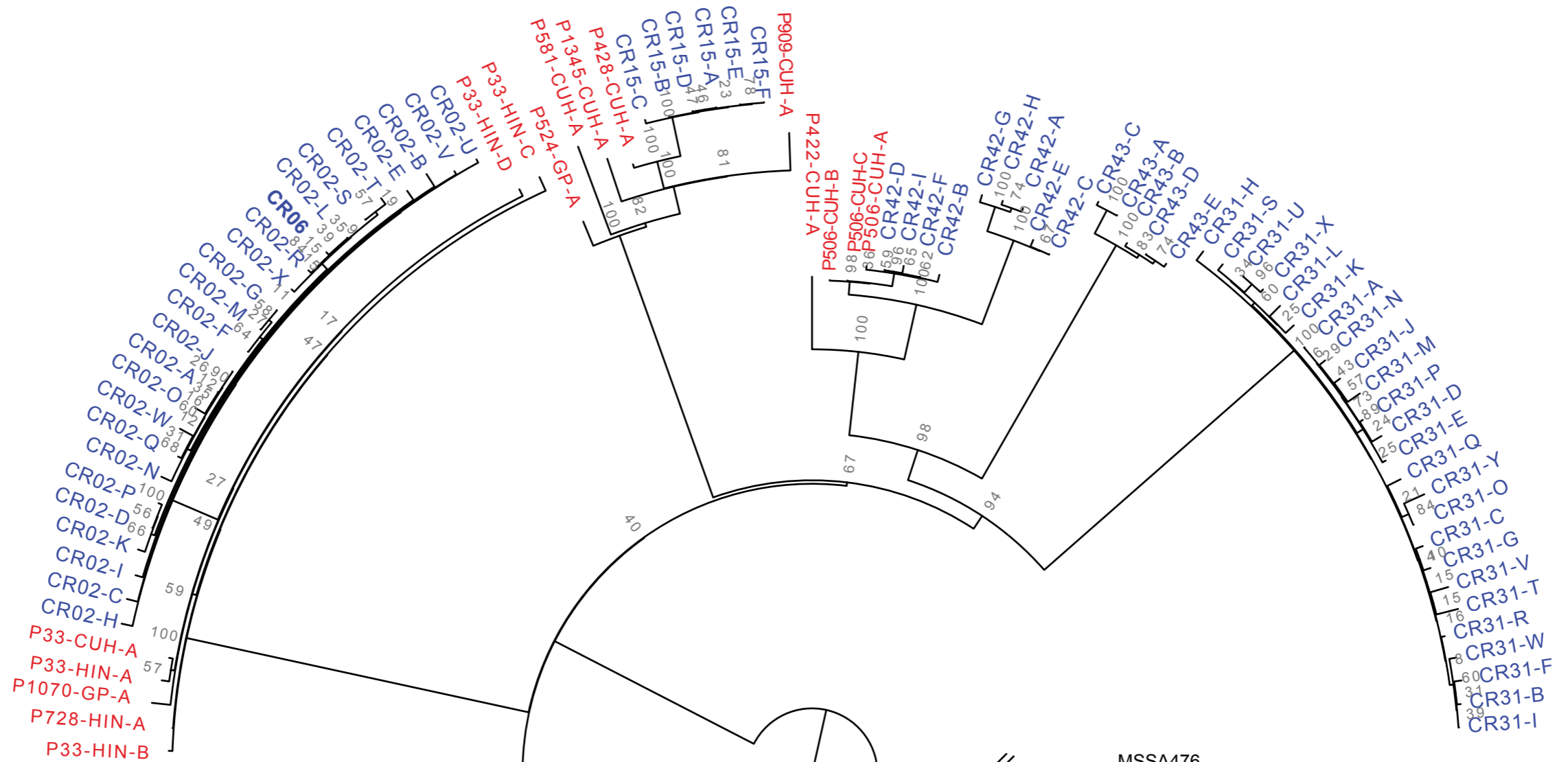

~18SNPs

Supplement: Additional file 3: — Phylogeny of CC22 MRSA in the Cambridge LTCF with bootstrap values. Bootstrap values for branches are shown in grey. (PDF 224 kb) [file 13073_2016_353_MOESM3_ESM.pdf]

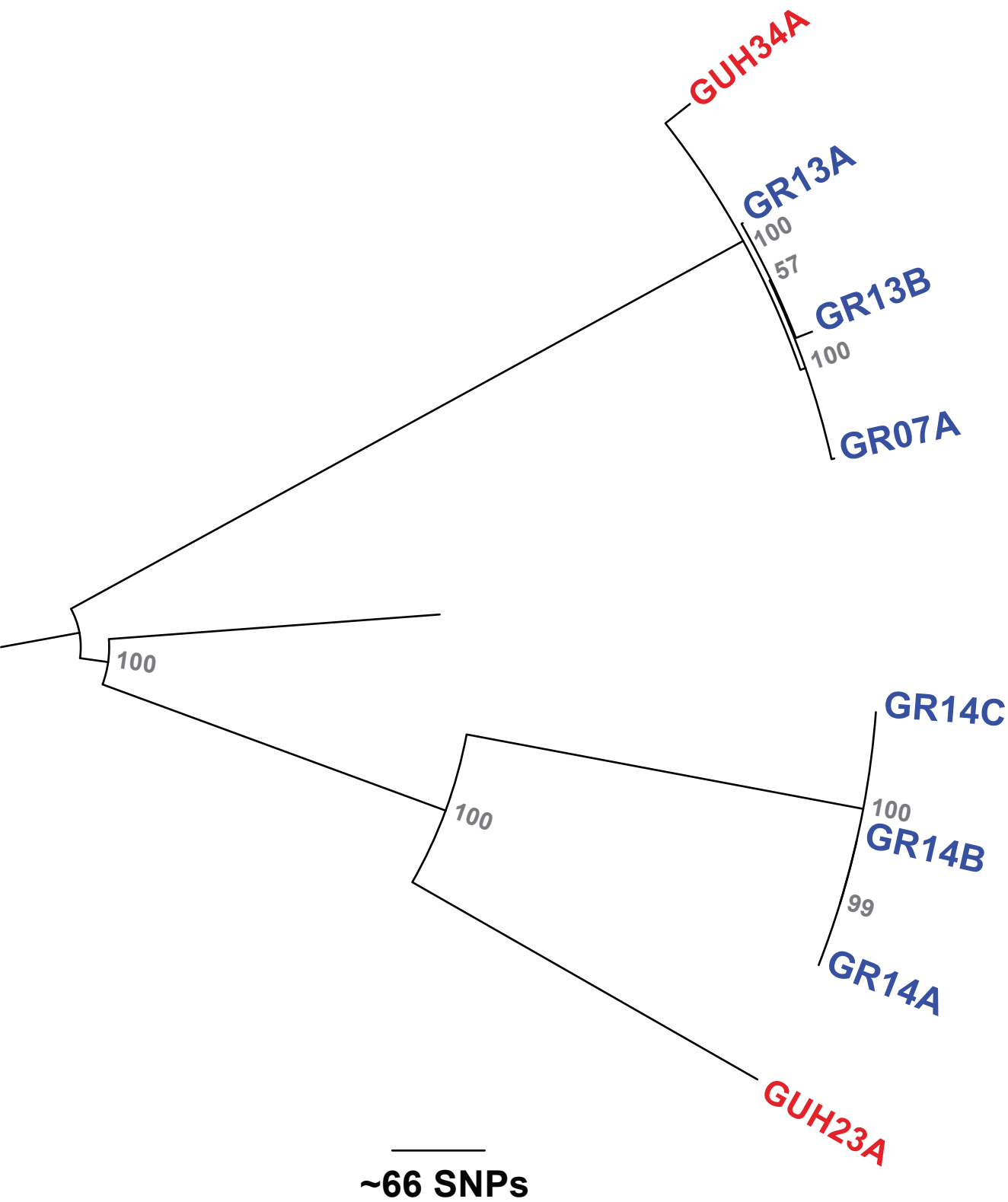

Supplement: Additional file 4: — Phylogeny of CC5 MRSA in the Galway study. Maximum likelihood tree generated from core genome SNPs of CC5 MRSA isolates from the Galway LTCF residents (blue) and isolates from Galway University Hospital (red). Bootstrap values for branches are shown in grey. (PDF 266 kb) [file 13073_2016_353_MOESM4_ESM.pdf]

**a**

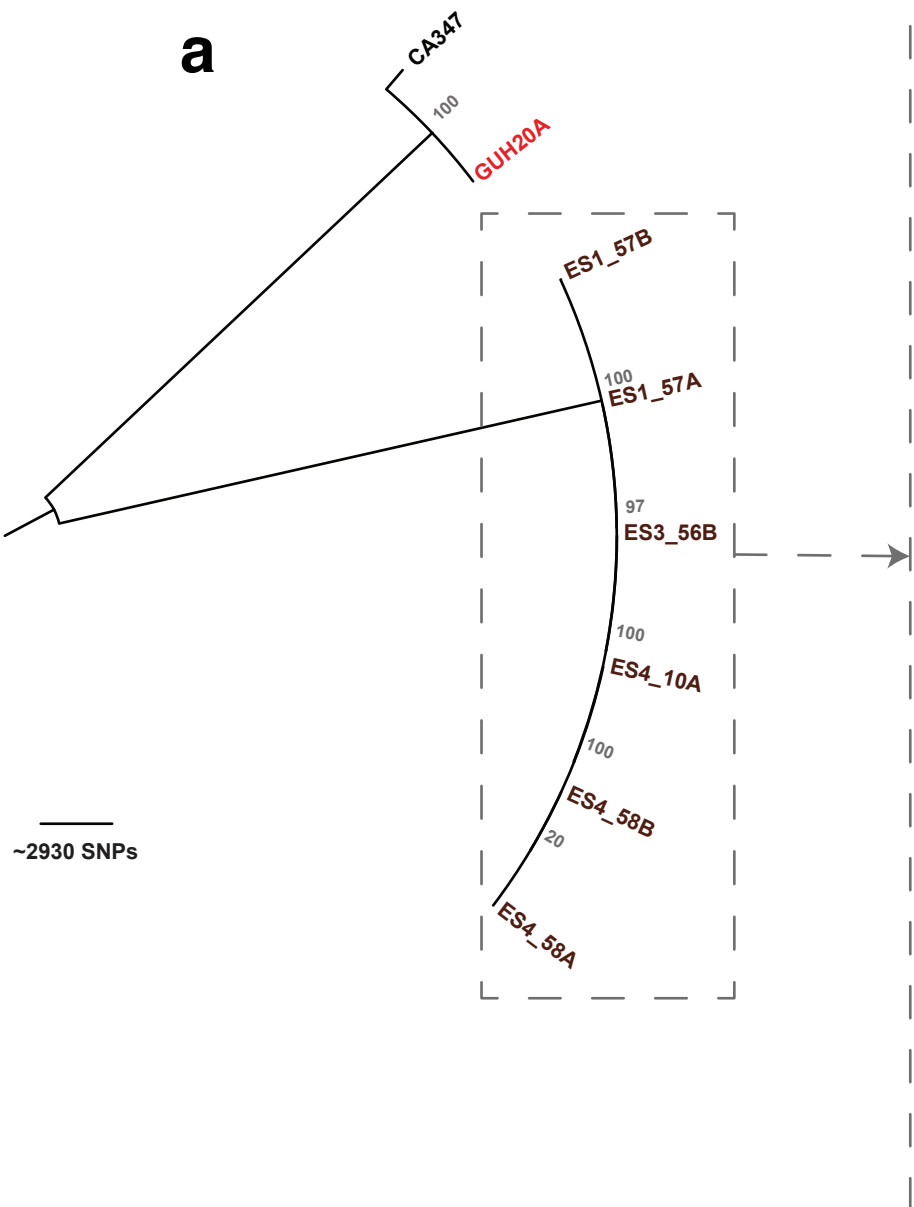

**b**

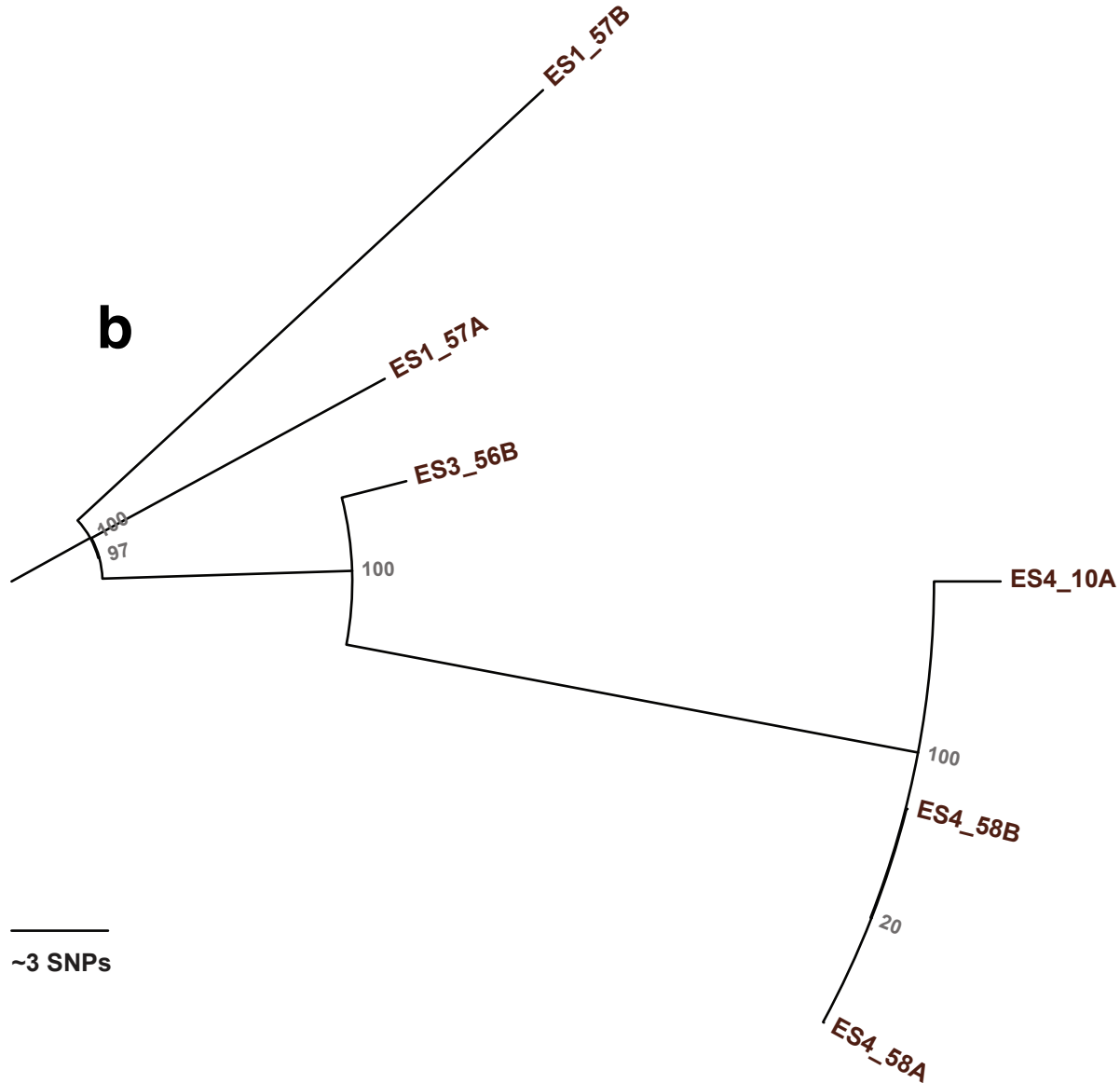

Supplement: Additional file 5: — Phylogeny of ST45 MRSA in the Galway study. a Maximum likelihood tree generated from core genome SNPs of ST45 MRSA isolates from the Galway LTCF environment (black) and the isolate from Galway University Hospital (red). b Zoomed in view of the isolates from the Galway LTCF. Bootstrap values for branches are shown in grey. (PDF 23 kb) [file 13073_2016_353_MOESM5_ESM.pdf]
